# Serum and Synovial Markers in Patients with Rheumatoid Arthritis and Periprosthetic Joint Infection

**DOI:** 10.3390/jpm12050810

**Published:** 2022-05-17

**Authors:** Yi Ren, Lara Biedermann, Clemens Gwinner, Carsten Perka, Arne Kienzle

**Affiliations:** 1 Center for Musculoskeletal Surgery, Clinic for Orthopedics, Charité University Hospital, 10117 Berlin, Germany; yi.ren@charite.de (Y.R.); lara.biedermann@charite.de (L.B.); clemens.gwinner@charite.de (C.G.); carsten.perka@charite.de (C.P.); 2 Berlin Institute of Health, Charité—Universitätsmedizin Berlin, BIH Biomedical Innovation Academy, BIH Charité Clinician Scientist Program, Charitéplatz 1, 10117 Berlin, Germany

**Keywords:** periprosthetic joint infection, rheumatoid arthritis, arthroplasty, total knee replacement, total hip replacement

## Abstract

Current diagnostic standards for PJI rely on inflammatory markers that are typically elevated in autoimmune diseases, thus making the diagnosis of PJI in patients with rheumatoid arthritis and joint replacement particularly complicated. There is a paucity of data on differentiating PJI from rheumatoid arthritis in patients with previous arthroplasty. In this study, we retrospectively analyzed the cases of 17 patients with rheumatoid arthritis and 121 patients without rheumatoid disease who underwent surgical intervention due to microbiology-positive PJI of the hip or knee joint. We assessed clinical patient characteristics, laboratory parameters, and prosthesis survival rates in patients with and without rheumatoid arthritis and acute or chronic PJI. ROC analysis was conducted for the analyzed parameters. In patients with chronic PJI, peripheral blood CRP (*p* = 0.05, AUC = 0.71), synovial WBC count (*p* = 0.02, AUC = 0.78), synovial monocyte cell count (*p* = 0.04, AUC = 0.75), and synovial PMN cell count (*p* = 0.02, AUC = 0.80) were significantly elevated in patients with rheumatoid arthritis showing acceptable to excellent discrimination. All analyzed parameters showed no significant differences and poor discrimination for patients with acute PJI. Median prosthesis survival time was significantly shorter in patients with rheumatoid arthritis (*p* = 0.05). In conclusion, routinely used laboratory markers have limited utility in distinguishing acute PJI in rheumatoid patients. In cases with suspected chronic PJI but low levels of serum CRP and synovial cell markers, physicians should consider the possibility of activated autoimmune arthritis.

## 1. Introduction

PJI is a major complication following joint replacement occurring in 1–5% of patients with primary arthroplasties [1,2]. Depending on the duration of symptoms, PJI is classified as acute or chronic. While the exact cutoff value is of ongoing debate, acute PJI is commonly defined as an infection with symptom duration ≤ 4 weeks [3,4]. In chronic PJI, symptoms have been present for > 4 weeks and may be the result of a low-virulence organism [5]. In both cases, adequate surgical treatment of PJI is mandatory to achieve a successful, infection-free outcome [6,7]. While treatment with debridement and implant retention can be an effective therapy for acute PJI, one- or two-stage exchange surgery may be required in chronic PJI [8]. In any of these cases, treatment is an enormous burden for patients [9]. In addition to surgical intervention, exchange arthroplasty can significantly impact joint function, cause pain, and has an increased risk of prosthesis failure [10,11,12].

Attending physicians are often challenged by the need to accurately diagnose PJI within a short time frame to be able to decide upon the necessary treatment strategy. Despite significant progress in recent years, no agreed-upon gold standard for the diagnosis of PJI exists [13]. Besides clinical presentation, diagnosis usually relies upon laboratory diagnostics using peripheral blood as well as synovial fluid. The markers routinely used are WBC count and serum CRP, as well as synovial WBC count and PMN cell percentage [14,15]. Depending on national, regional, or hospital-specific guidelines and standards, additional testing for leukocyte esterase, alpha-defensin, D-dimer, erythrocyte sedimentation rate, and synovial CRP may be performed. Additionally, microbiological culture is essential but not feasible in an acute setting due to culture time [16]. In some cases, microbiological culture may be negative despite the presence of PJI [17,18].

While both the 2018 Definition of Periprosthetic Hip and Knee Infection by Parvizi et al. and the EBJIS definition of PJI are reliable clinical guidelines for most affected patients, the criteria listed may not be feasible for all patients [14,15]. In particular, diagnosis of both acute and chronic PJI is complicated in patients with rheumatoid arthritis where aseptic joint inflammation causes similar clinical and laboratory presentation. Qin et al. recently demonstrated that commonly used laboratory markers of non-operated rheumatoid arthritis patients do not differ significantly to those of patients with chronic PJI [19]. Patients with active rheumatoid arthritis of the operated joint are always scored to be likely affected by PJI. There is a paucity of data on differentiating PJI from rheumatoid arthritis in patients with previous knee or hip arthroplasty. While PJI cannot be ruled out with current diagnostic standards, a more detailed understanding of the relevant serum and synovial marker levels is necessary to personalize diagnostics and avoid unnecessary surgical intervention.

In this study, we retrospectively analyzed the cases of 17 patients with rheumatoid arthritis and 121 patients with no diagnosed rheumatoid disease who underwent surgical intervention due to microbiology-positive PJI of the hip or knee joint. This is the first study to evaluate differences in serum and synovial fluid markers in patients affected by this pathology.

## 2. Materials and Methods

### 2.1. Patients

This study was approved by the Charité University hospital ethics board (EA2/083/19) and was completed in accordance with the Declaration of Helsinki.

All patients receiving total knee or hip replacement exchange surgery due to acute or chronic PJI between 2013 and 2021 at the Charité university hospital in Berlin, Germany were retrospectively analyzed in this study. Patients were treated in a specialized department using a centralized and interdisciplinary treatment approach. In total, we analyzed patient files of 138 patients.

Inclusion criteria were a previously implanted knee or hip replacement and diagnosed PJI. As rheumatoid arthritis and PJI share clinical and paraclinical features, PJI was defined according to modified EBJIS criteria [20]: microbiological growth in synovial fluid, two or more tissue samples (for highly virulent organisms or in patients being treated with antibiotics, one positive sample confirmed infection), or sonication fluid (>50 CFU/mL) and at least one of the following criteria: (1) prevalence of a sinus tract or purulence around a component; (2) >2000 leukocytes/µL or >70% granulocytes in the synovial fluid; or (3) histology of intra-operatively acquired tissue Krenn and Morawietz type II or type III [21]. Acute PJI was defined as an infection within 4 weeks after primary arthroplasty surgery or acute onset of PJI-related symptoms less than 4 weeks before diagnosis and treatment of PJI. Symptom onset >4 weeks was classified as chronic PJI.

Rheumatoid arthritis was diagnosed prior to occurrence of PJI by a board-certified rheumatologist according to the ACR/EULAR Classification Criteria [22]. All patients were actively treated by a rheumatologist.

Patients who met one or more of the following criteria were excluded from this study: (1) culture-negative patients meeting EBJIS criteria for PJI; or (2) primary knee or hip joint infection without prosthesis. There were no further exclusions.

The enrolled patient population was divided into two groups based on whether patients diagnosed with rheumatoid disease (group A) or not (group B). Both groups were subdivided into acute and chronic cases: A1, acute cases with immune disorders; A2, chronic cases with immune disorders; B1, acute cases without immune disorders; B2, chronic cases without immune disorders.

Besides clinical and paraclinical examination, we assessed demographic data including age, BMI, ASA score, the number of prior surgeries on the affected knee or hip, pathological classification of tissue specimens, and laboratory results.

### 2.2. Statistical Analysis

All data were collected and recorded in Microsoft^®^ Excel^®^ 2016 (version 2111 Build 16.0.14701.20240, Microsoft, Redmond, WA USA). Continuous data were presented as median and IQR and analyzed using Student’s t test or Mann–Whitney U test where applicable. Data between two groups were compared using chi-square test. Optimal cut-off values were determined using the Youden index (J) method (maximal value of “sensitivity + specificity-1”) [23]. Based on cut-offs, sensitivity and specificity were defined and NPV, PPV, ROC, and AUC determined. Survival analysis was presented through Kaplan–Meier survival curves. All statistical analyses and plots were analyzed using R software (version: 3.6.3. R Development Core Team, Vienna, Austria).

## 3. Results

### 3.1. Patient Characteristics

Patient characteristics are outlined in Table 1. In total, 138 patients were enrolled in this study: 17 patients with rheumatoid arthritis and PJI (group A) and 121 patients without rheumatoid arthritis and PJI (group B). Of the patients included in our analysis, 76 were male (group A: 12; group B: 64) and 62 were female (group A: 5; group B: 57). Average patient age was 72.94 ± 7.10 years in group A and 69.07 ± 10.83. Mean BMI was 29.83 ± 6.97 for group A and 30.59 ± 5.82 for group B. Most patients had an ASA score of 2 (17.65% group A; 56.20% group B) or 3 (70.59% group A; 36.36% group B). Acute PJI occurred in 9 (52.94%; group A) and 54 (44.63%; group B) patients, and chronic PJI in 8 (47.06%; group A) and 67 (55.37%; group B) patients. Most patients had more than one revision surgery prior to PJI (70.59% in group A; 61.98% in group B). No significant differences for any of the analyzed parameters were found.

### 3.2. Pathology and Microbiology

Pathology results indicated an infection (Krenn and Morawietz score of 2 or 3) in 88.24% of the patients with rheumatoid arthritis (group A) and in 77.69% of the patients without rheumatoid arthritis (group B; *p* = 0.32). Of these, 66.67% (group A) and 55.32% (group B) had a low-grade infection and 33.33% (group A) and 44.68% (group B) had a high-grade infection (*p* = 0.41). The remaining patients had a Krenn and Morawietz score of 1 or 4: 11.76% in group A and 22.31% in group B. In none of the patients analyzed was a sinus tract prevalent.

For all patients, synovial fluid samples were analyzed for pathogens (Table 2). In both groups, *Staphylococcus aureus* (47.06% in group A, 33.06% in group B) followed by *Staphylococcus epidermidis* (35.29% in group A, 19.83% in group B) had the highest incidence rate.

### 3.3. Laboratory

Peripheral blood CRP concentration and WBC numbers as well as synovial fluid cell counts were analyzed for all patients. For acute PJI, no significant difference between patients with (group A1) and without rheumatoid arthritis (group B1) were found (Table 3): Median CRP was 88.00 and 129.45 mg/L (*p* = 0.92), WBC count 9.13 and 9.93 cells/nL (*p* = 0.30), synovial WBC 60.75 and 48.92 cells/nL (*p* = 0.54), and synovial PMN cell count 55.89 and 48.24 cells/nL (*p* = 0.74), respectively. All parameters analyzed showed high variability.

In patients with chronic PJI, peripheral blood CRP (group A2: 43.25 versus B2: 18.80 mg/L; *p* = 0.05), synovial WBC count (group A2: 34.68 versus B2: 8.33 cells/nL; *p* = 0.02), synovial monocyte cell count (group A2: 2.27 versus B2: 0.79 cells/nL; *p* = 0.04), and synovial PMN cell count (group A2: 33.36 versus B2: 6.13 cells/nL; *p* = 0.02) were significantly elevated in patients with rheumatoid arthritis (Table 3). In contrast, peripheral blood WBC count did not differ significantly (group A2: 6.86 versus B2: 7.45 cells/nL; *p* = 0.75).

**Figure 1:**
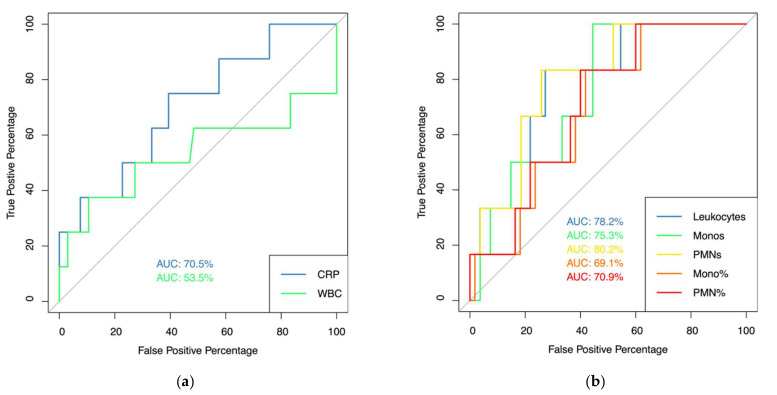
AUC Analysis. (**a**) AUC analysis for serum CRP (blue line) and peripheral blood WBC count (green line) in patients with chronic PJI; (**b**) AUC analysis for synovial fluid WBC count (blue line), monocyte cell count (green line), PMN cell count (yellow line), synovial percentage of monocytes (orange line), and percentage of PMN cell count (red line).

ROC analysis was conducted for the analyzed parameters: AUC, best cut-off values, sensitivity, specificity, and NPV and PPV are listed in Table 4. All analyzed parameters showed poor discrimination for patients with acute PJI. Conversely, in patients with chronic PJI serum CRP levels (AUC = 0.71), synovial WBC count (AUC = 0.78), synovial monocyte cell count (AUC = 0.75), and synovial percentage of PMN cell count (AUC = 0.71) showed acceptable discrimination and synovial PMN cell count (AUC = 0.80) showed excellent discrimination (Figure 1). While for any of these parameters, sensitivity and NPV was above 75% and 95%, respectively, specificity and PPV only ranged from 55% to 74% and 18% to 26%, respectively.

### 3.4. Prosthesis Survival

**Figure 2:**
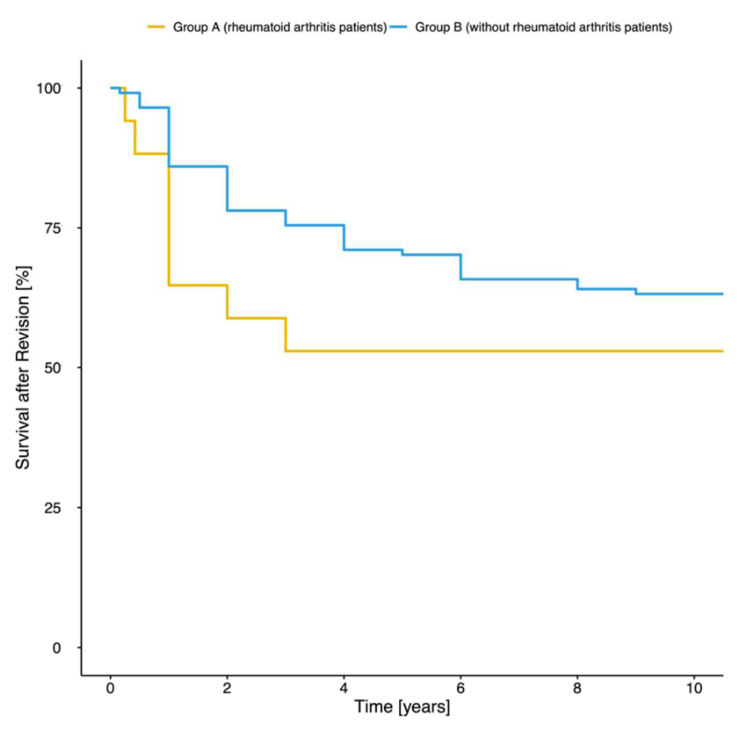
Prosthesis survival rate. Diagnosed recurrent PJI or aseptic loosening was classified as prosthesis failure. After 9 years, 47.06% of patients with rheumatoid arthritis (blue line) and 21.93% of patients without rheumatoid arthritis (yellow line) had suffered from prosthesis failure.

Risk for prosthesis failure due to recurrent PJI or aseptic loosening (Figure 2) was significantly elevated in patients with rheumatoid arthritis (prosthesis survival rate in group A: 78.07% versus group B: 52.94%; *p* = 0.03). Additionally, median prosthesis survival times were significantly shorter in group A (median: 1 year, IQR: 1.00–3.00 years) compared to group B (median: 2 years, IQR: 1.75–4.00 years; *p* = 0.05).

## 4. Discussion

In this study, we analyzed differences in clinical patient characteristics, laboratory parameters, and prosthesis survival rates in patients with and without rheumatoid arthritis and acute or chronic PJI. Additionally, we retrospectively evaluated the capability of laboratory markers to distinguish these patient groups. Long-term revision arthroplasty failure rate was significantly elevated in patients with rheumatoid arthritis and PJI compared to patients without autoimmune disease.

In both acute and chronic PJI, attending physicians are challenged to accurately confirm diagnosis in patients that are presenting with clinical features of PJI. Similar clinical and laboratory features of aseptic joint inflammation in patients with rheumatoid arthritis and arthroplasty significantly complicate the diagnosis of PJI. All patients with symptoms of active autoimmune arthritis after primary arthroplasty are classified as PJI-likely cases [14,15]. Inherently, investigations are limited to positive cases as PJI-negative cases in patients with active rheumatoid arthritis do not exist per definition. Commonly, diagnosis relies upon peripheral blood WBC count and serum CRP, as well as synovial WBC count and PMN cell percentage [13,14,15]. Establishing the diagnosis is challenging as guidelines were derived from PJI patients without rheumatoid arthritis [24]. Previous research reported the risk of infection in patients with rheumatoid arthritis to be significantly increased [7,25], potentially due to anti-rheumatic immunosuppressive therapy [26]. However, Trikha et al. found rheumatoid arthritis not to be an independent risk-factor for PJI in a murine model [27], suggesting PJI may be falsely diagnosed in some patients. Thus, in our study, only culture-positive patients were included to avoid analysis of false-positive cases.

To initiate treatment and avoid short- and long-term complications such as sepsis, recurrent PJI or aseptic loosening, a diagnosis is often needed in a short time frame. While microbiological culture is essential, it is not feasible in an acute setting due to culture time [16] and may be negative despite the presence of PJI [17,18]. In our study, we did not find good discriminatory power for peripheral blood WBC counts, serum CRP, synovial WBC count, synovial PNM cell count, synovial percentage of PNM cells, or synovial percentage of monocytes in acute PJI. While discriminatory power for these parameters was good to excellent in chronic PJI, specificity and PPV were only between 55% and 74% and 18% and 26%, respectively. Novel diagnostic serum and synovial markers such as alpha-defensin, soluble tumor necrosis factor receptor, and B-cell activating factor, as well as technologies such as next-generation sequencing, promise to improve current standards [18,28,29] and could especially benefit rheumatoid arthritis patients.

Due to the immediate severe impact on patients’ life quality and to avoid unnecessary surgery, particular consideration must be given to false-positive diagnoses [30,31]. Rheumatoid arthritis patients are especially at risk as improvement of quality of life has been found to be poorer compared to patients with osteoarthritis after primary total joint replacement [32]. In our study, we found prosthesis failure rates after revision arthroplasty to be significantly elevated in patients with rheumatoid arthritis, further stressing the need for a more personalized diagnostic and therapeutic approach in these patients. Similarly, prosthesis survival rates after revision arthroplasty have been found to be significantly decreased in non-rheumatoid arthritis patients [11,12,33].

Limitations of the current study include the heterogeneity of the analyzed population, the retrospective study design, the analyzed rheumatoid arthritis cohort size, and the exclusion of potentially PJI-positive but culture-negative cases with potential subsequent statistical bias.

In conclusion, the current guidelines and routinely used laboratory markers have limited utility in distinguishing acute PJI in rheumatoid patients. In cases with suspected chronic PJI but low levels of serum CRP and synovial cell markers, physicians should consider the possibility of activated autoimmune arthritis. The observed elevated prosthesis failure rate in these patients stresses the need for novel diagnostic markers and a more personalized diagnostic and therapeutic approach for affected patients.

## Figures and Tables

**Table 1:** Patient Characteristics.

	Group A (Rheumatoid Arthritis Patients)	Group B (without Rheumatoid Arthritis Patients)	*p* Value
Sex			
Male [# (%)]	12 (70.59%)	64 (52.89%)	0.17
Female [# (%)]	5 (29.41%)	57 (47.11%)	
BMI [kg/m^2^]	29.83 ± 6.97	30.59 ± 5.82	0.69
Age [years]	72.94 ± 7.10	69.07 ± 10.83	0.06
PJI onset			
Acute [# (%)]	9 (52.94%)	54 (44.63%)	0.52
Chronic [# (%)]	8 (47.06%)	67 (55.37%)	
ASA score			
1 [# (%)]	0 (0.00%)	2 (1.65%)	0.06
2 [# (%)]	3 (17.65%)	68 (56.20%)	
3 [# (%)]	12 (70.59%)	44 (36.36%)	
4 [# (%)]	1 (5.88%)	4 (3.31%)	
5 [# (%)]	0 (0.00%)	1 (0.83%)	
Number of prior revision surgeries			
One [# (%)]	5 (29.41%)	46 (38.02%)	0.49
More than one [# (%)]	12 (70.59%)	75 (61.98%)	

#, number of patients.

**Table 2:** Pre- and perioperative pathogens.

Pathogen	Group A (Rheumatoid Arthritis Patients)	Group B (without Rheumatoid Arthritis Patients)
*Staphylococcus aureus*	8 (47.06%)	40 (33.06%)
*Staphylococcus epidermidis*	6 (35.29%)	24 (19.83%)
*Cutibacterium acnes*	-	12 (9.91%)
*Enteroccocus faecalis*	1 (5.88%)	10 (8.26%)
*Streptococcus anginosus*	1 (5.88%)	2 (1.65%)
*Streptococcus dysgalactiae*	1 (5.88%)	8 (6.61%)
*Escherichia coli*	-	7 (5.79%)
*Staphylococcus hominis*	-	8 (6.61%)
*Candida albicans*	-	1 (0.83%)
*Candida parapsilosis*	-	2 (1.65%)
*Cutibacterium avidum*	-	1 (0.83%)
*Staphylococcus capitis*	-	2 (1.65%)
*Streptococcus agalactiae*	-	1 (0.83%)
*Streptococcus mitis*	-	1 (0.83%)
*Streptococcus pyogenes*	-	1 (0.83%)
*Streptococcus pneumoniae*	-	1 (0.83%)

**Table 3:** Laboratory results before prosthesis explantation.

	Group A1 (Rheumatoid Arthritis Patients; *n* = 9)		Group B1 (without Rheumatoid Arthritis Patients; *n* = 54)			
	Median	IQR	Median	IQR	W	*p* Value
**Acute PJI**						
Serum CRP [mg/L]	88.00	86.90–256.20	129.45	72.03–244.22	237	0.92
Peripheral blood WBC count [cells/nL]	9.13	6.17–12.03	9.93	7.22–14.22	190	0.30
Synovial WBC count [cells/nL]	60.75	54.72–118.06	48.92	33.58–197.56	178	0.54
Synovial monocyte cell count [cells/nL]	6.69	2.21–11.43	3.97	2.05–13.85	136	0.93
Synovial PMN cell count [cells/nL]	55.89	48.41–86.94	48.24	31.30–160.93	144	0.74
Synovial percentage of monocytes [%]	0.11	0.04–0.12	0.09	0.05–0.16	120	0.69
Synovial percentage of PMN cells [%]	0.89	0.88–0.96	0.91	0.84–0.95	149	0.62
	**Group A2** **(Rheumatoid Arthritis Patients;** ***n* = 8)**		**Group B2** **(without Rheumatoid Arthritis Patients;** ***n* = 67)**			
	**Median**	**IQR**	**Median**	**IQR**	**W**	***p* Value**
**Chronic PJI**						
Serum CRP [mg/L]	43.25	25.02–145.00	18.80	6.45–47.15	372	0.05
Peripheral blood WBC count [cells/nL]	6.86	5.16–10.81	7.45	6.25–8.39	245	0.75
Synovial WBC count [cells/nL]	34.68	23.06–103.17	8.33	0.86–23.37	258	0.02
Synovial monocyte cell count [cells/nL]	2.27	1.16–13.5	0.79	0.33–2.28	244	0.04
Synovial PMN cell count [cells/nL]	33.36	20.48–70.75	6.13	0.43–16.68	260	0.02
Synovial percentage of monocytes [%]	0.10	0.05–0.15	0.23	0.08–0.43	102	0.13
Synovial percentage of PMN cells [%]	0.90	0.85–0.95	0.77	0.55–0.91	234	0.09

**Table 4:** Diagnostic value analysis.

	Cut-Off	Sensitivity	Specificity	NPV	PPV	AUC	AUC CI
**Acute PJI**							
Serum CRP [mg/L]	107.65	33.30%	38.90%	77.80%	8.30%	0.51	0.31–0.70
Peripheral Blood WBC count [cells/nL]	13.36	16.80%	63.00%	79.10%	8.40%	0.61	0.43–0.78
Synovial WBC count [cells/nL]	43.18	100%	35.90%	100%	24.20%	0.57	0.41–0.73
Synovial monocyte cell count [cells/nL]	2.06	100%	26.30%	100%	20.00%	0.51	0.30–0.71
Synovial PMN cell count [cells/nL]	37.79	100%	31.60%	100%	21.20%	0.54	0.37–0.70
Synovial percentage of monocytes [%]	10.07	57.10%	63.20%	88.90%	22.20%	0.45	0.20–0.69
Synovial percentage of PMN cells [%]	0.90	100%	9.30%	100%	15.60%	0.44	0.20–0.69
**Chronic PJI**							
Serum CRP [mg/L]	29.05	75.00%	60.60%	95.20%	18.80%	0.71	0.50–0.90
Peripheral Blood WBC count [cells/nL]	5.495	62.50%	10.60%	70.00%	7.80%	0.54	0.23–0.83
Synovial WBC count [cells/nL]	19.48	83.30%	72.70%	97.60%	25.00%	0.78	0.61–0.95
Synovial monocyte cell count [cells/nL]	0.83	100%	55.60%	100%	20.00%	0.75	0.58–0.92
Synovial PMN cell count [cells/nL]	16.18	83.30%	74.10%	97.60%	26.30%	0.80	0.63–0.96
Synovial percentage of monocytes [%]	14.70	16.70%	41.80%	82.10%	3.00%	0.69	0.50–0.87
Synovial percentage of PMN cells [%]	85.30	83.30%	73.00%	97.10%	18.50%	0.71	0.52–0.90

## Data Availability

All data presented in this study are available on request from the corresponding author.

## Abbreviations

American Society of Anesthesiologists, ASA; area under curve, AUC; body mass index, BMI; C-reaction protein, CRP; interquartile range, IQR; negative predictive values, NPV; periprosthetic joint infection, PJI; polymorphonuclear, PMN; positive predictive values, PPV; receiver operating curve, ROC; white blood cell, WBC.
